# Supplementary material for: Mutation of an insulin-sensitive Drosophila insulin-like receptor mutant requires methionine metabolism reprogramming to extend lifespan
Source: bioRxiv. 2025 Mar 4:2025.02.28.640731. Preprint. [Version 1] doi: 10.1101/2025.02.28.640731 (PMC11908128; doi:10.1101/2025.02.28.640731)
Supplement: Supplement 1 [file NIHPP2025.02.28.640731v1-supplement-1.pdf]

bioRxiv preprint doi: https://doi.org/10.1101/2025.02.28.640731; this version posted March 4, 2025. The copyright holder for this preprint (which was not certified by peer review) is the author/funder, who has granted bioRxiv a license to display the preprint in perpetuity. It is made available under aCC-BY 4.0 International license.

Supplementary Figure 1. ***Metabolomic profiles of dInr^{E19}/dInr^{74} and dInr^{353}/dInr^{wt}*****.** (**A**) Principal component analysis of metabolomic profiling in *Type I dInr^{E19}/dInr^{74} and Type II dInr*^{353}/*dInr*^{wt} genotypes. (**B**) Relative levels of metabolites (cystathionine and methionine sulfoxide) significantly altered in *dInr*^{353}/*dInr*^{wt} (Type **II**) genotype. (**C**) Relative levels of metabolites (hypotaurine, taurine, adenosine, reduced glutathione) significantly altered in *dInr^{E19}/dInr^{74}* (Type **I**) genotype. (**D**) Relative levels of metabolites (sarcosine) significantly altered in both *Type I dInr^{E19}/dInr^{74} and Type II dInr*^{353}/*dInr*^{wt} genotypes.

Sup. Figure 1

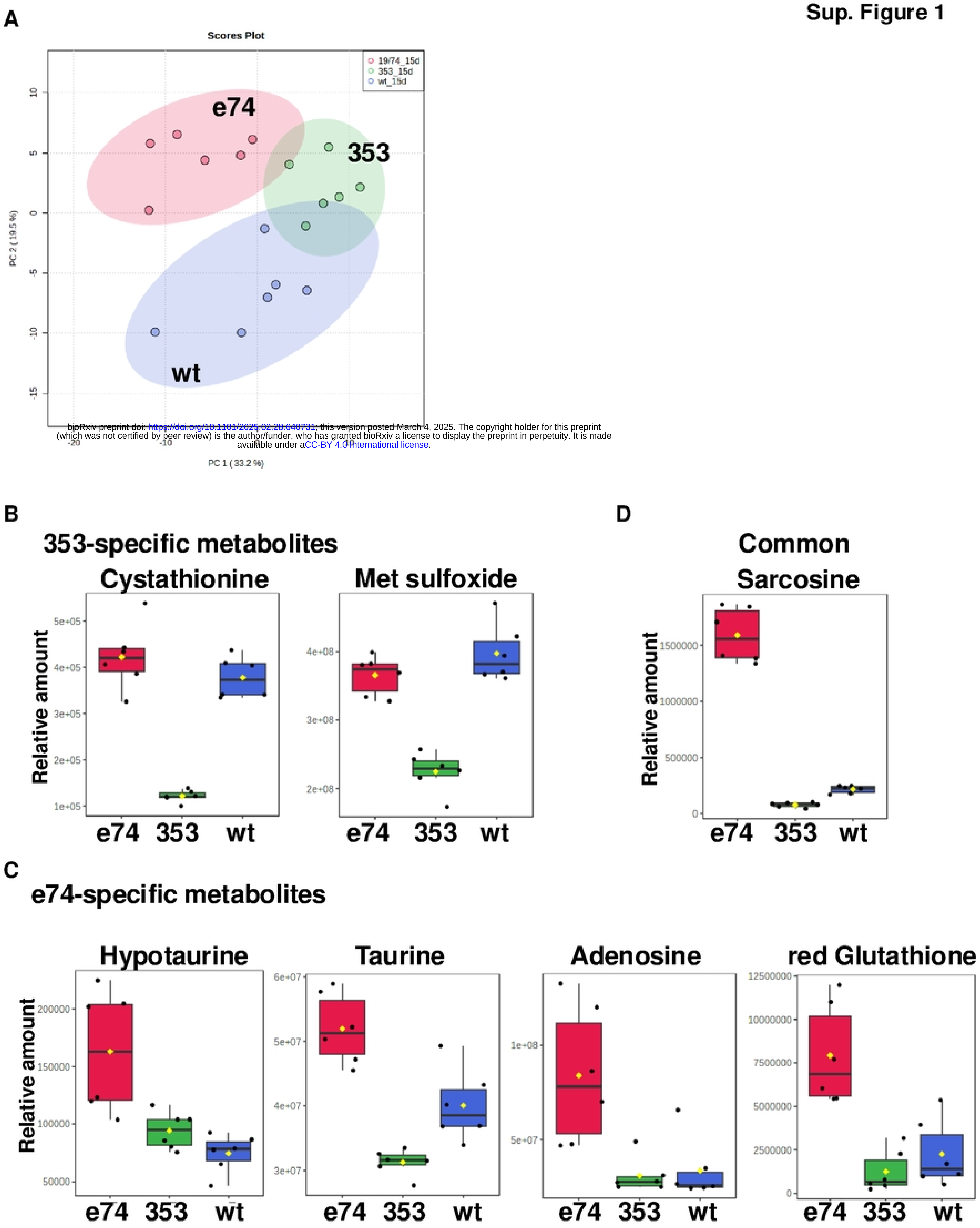

bioRxiv preprint doi: https://doi.org/10.1101/2025.02.28.640731; this version posted March 4, 2025. The copyright holder for this preprint (which was not certified by peer review) is the author/funder, who has granted bioRxiv a license to display the preprint in perpetuity. It is made available under aCC-BY 4.0 International license.
